# Revealing the Characteristics of the Antarctic Snow Alga *Chlorominima collina* gen. et sp. nov. Through Taxonomy, Physiology, and Transcriptomics

**DOI:** 10.3389/fpls.2021.662298

**Published:** 2021-06-07

**Authors:** Francisca E. Gálvez, Mónica Saldarriaga-Córdoba, Pirjo Huovinen, Andrea X. Silva, Iván Gómez

**Affiliations:** ^1^Instituto de Ciencias Marinas y Limnológicas, Facultad de Ciencias, Universidad Austral de Chile, Valdivia, Chile; ^2^Centro FONDAP de Investigación en Dinámica de Ecosistemas Marinos de Altas Latitudes (IDEAL), Valdivia, Chile; ^3^Centro de Investigación en Recursos Naturales y Sustentabilidad (CIRENYS), Universidad Bernardo O’Higgins, Santiago, Chile; ^4^Instituto de Ciencias Ambientales y Evolutivas, Facultad de Ciencias, Universidad Austral de Chile, Valdivia, Chile; ^5^AUSTRAL-omics, Vicerrectoría de Investigación, Desarrollo y Creación Artística, Universidad Austral de Chile, Valdivia, Chile

**Keywords:** Antarctic, *Chlorominima*, polyphasic approach, psychrophilic, snow algae, cysts, transcriptome

## Abstract

Snow algae play crucial roles in cold ecosystems, however, many aspects related to their biology, adaptations and especially their diversity are not well known. To improve the identification of snow algae from colored snow, in the present study we used a polyphasic approach to describe a new Antarctic genus, *Chlorominima* with the species type *Chlorominima collina*. This new taxon was isolated of colored snow collected from the Collins Glacier (King George Island) in the Maritime Antarctic region. Microscopy revealed biflagellated ellipsoidal cells with a rounded posterior end, a C-shaped parietal chloroplast without a pyrenoid, eyespot, and discrete papillae. Several of these characteristics are typical of the genus *Chloromonas*, but the new isolate differs from the described species of this genus by the unusual small size of the cells, the presence of several vacuoles, the position of the nucleus and the shape of the chloroplast. Molecular analyzes confirm that the isolated alga does not belong to *Chloromonas* and therefore forms an independent lineage, which is closely related to other unidentified Antarctic and Arctic strains, forming a polar subclade in the *Stephanosphaerinia* phylogroup within the Chlamydomonadales. Secondary structure comparisons of the ITS2 rDNA marker support the idea that new strain is a distinct taxon within of *Caudivolvoxa*. Physiological experiments revealed psychrophilic characteristics, which are typical of true snow algae. This status was confirmed by the partial transcriptome obtained at 2°C, in which various cold-responsive and cryoprotective genes were identified. This study explores the systematics, cold acclimatization strategies and their implications for the Antarctic snow flora.

## Introduction

The cryospheric biome is dominated by highly specialized microorganisms that thrive under extreme low temperatures at the interface between snow/ice and liquid water (Anesio and Laybourn-Parry, 2012). The basis of these microbial communities, and hence the precursors of inorganic carbon fixation and primary source of macromolecules, are snow and ice algae, and cyanobacteria (Boetius et al., 2015; Anesio et al., 2017). Especially during the thaw season, these algae actively fix carbon (Yallop et al., 2012; Lutz et al., 2014). In fact, daily measurements of gas exchange in dense patches of snow algae have shown values of CO_2_ uptake close to 2,300 μmol m^–2^ day^–1^, indicating that summer snowfields can be surprisingly productive and even in some circumstances can be a significant CO_2_ sink (Williams et al., 2003; Hamilton and Havig, 2017). Snow algae play crucial ecological roles as foundation organisms sustaining a high diversity of heterotrophic micro-eukaryotes, bacteria, and archaea (Lutz et al., 2015; Havig and Hamilton, 2019). The biological interactions within these communities, especially mutualism, have enhanced resilience to changes in the snow environment (Krug et al., 2020). In fact, it has been suggested that such biological processes can promote horizontal exchange and recombination of genetic material, which enables the acquisition of new genes, enhancing diversity (Lyon and Mock, 2014; Liu et al., 2018).

The identification of snow algae had previously been limited to morphological observations of cysts of red snow that were recognized as zygotes of the algae *Chlamydomonas nivalis* (Bauer) Wille and *Chloromonas nivalis* (Chodat) Hoham and Mullet. Therefore, these species were recurrently associated with the global distribution of cysts, resulting in cosmopolitan species (Marchant, 1982; Gradinger and Nürnberg, 1996; Müller et al., 1998). In the case of green snow, the identification of vegetative cells, also was normally based only on microscopic observations (Kol, 1968; Ettl, 1970). This has probably challenged the comprehensive identification of cryosestic algae in the past. However, the use of polyphasic approaches that include the combined use of multi-gene analyses, light and electron microscopy, biochemical and physiological approaches (Pröschold and Leliaert, 2007), strain designations by culture collections, as well as a better access to samples from previously inaccessible regions, has allowed both the re-examination of many strains of snow algae and the description of novel lineages (Muramoto et al., 2010; Demchenko et al., 2012; Matsuzaki et al., 2014; Hoham and Remias, 2020). To the date, polyphasic analyses show that field-collected cysts identified as *Chloromonas nivalis* zygotes consist actually of multiple species (Matsuzaki et al., 2018, 2019). Extensive phylogenetic analyses performed on the *Chlamydomonas*–*Chloromonas* complex have shown that 21 taxa from cold environments occur in four clades (Hoham et al., 2002; Remias et al., 2010) being most of the snow algae included in the phylogroup *Chloromonadinia* (Nakada et al., 2008). Another clade composed of algae that cause reddish coloration includes the new genus *Sanguina* (Procházková et al., 2019a). Thus, it is possible to argue that diversity of snow algae has been underestimated and is just being revealed.

For Antarctica, since the first microscopic surveys (Fogg, 1967; Kol, 1971; Broady, 1996; Ling, 1996) it has been recognized that snow fields from continental and insular regions host abundant and diverse communities of snow algae. However, only few of these snow algae have been identified with certainty (Ling, 1996). In the case of the Maritime Antarctic region, especially the South Shetland Islands, the snow algae communities were dominated by species of the Trebouxiophyceae and Chlorophyceae classes, whose structure and functional traits are set basically by the marine influence, sources of eutrophication and color of bloom (Komárek and Komárek, 1999, 2001; Soto et al., 2020).

Considering that regional warming has become an important environmental threat in the Maritime Antarctic (Vaughan et al., 2003), the biological processes, and fate of snow microbial communities will be strongly impacted by melting (Boetius et al., 2015; Garcia-Lopez and Cid, 2017). For example, the Collins Glacier in King George Island, has lost 8.42% of its mass between 1,983 and 2,012, mostly in the north and central-west sectors (Cook, 2005; Simoes et al., 2015). In a first stage, snow algae could be favored in ablation zones as melting provides suitable environments for their development, thus reducing albedo and exacerbating melting (Huovinen et al., 2018). As a contribution to the knowledge of the Antarctic cryoflora, we isolated a new alga (*Chlorominima*) from the red snow of this glacier that is in close proximity to the coast. Snowfields close to marine bird and seal colonies are typical sites for the development of snow algae in Antarctica due to a high nutrient input (Kol and Flint, 1968; Komárek and Komárek, 1999; Remias et al., 2013). To continue monitoring the consequences of melting of the glaciers on the microbial diversity it is relevant not only to perform accurate taxonomic identifications, but also to characterize physiological responses and perform gene profiling to gain insight into the dynamic and functions of snow algae under changing environmental scenarios (Cvetkovska et al., 2017; Garcia-Lopez and Cid, 2017).

The present study aimed (i) to propose a new genus, with the species type *Chlorominima collina* identified through an integrative approach of cultures obtained from samples collected from the Collins Glacier, King George Island; and (ii) to combine physiological and transcriptional approaches to describe some key adaptive traits that allow this organism thriving under permanently low temperatures. Thus, this report connects systematics aspects with the ecology and functionality of an Antarctic snow alga from a region that is being impacted by multiple environmental changes.

## Materials and Methods

### Sample Collection, Isolation and Cultivation

**FIGURE 1:**
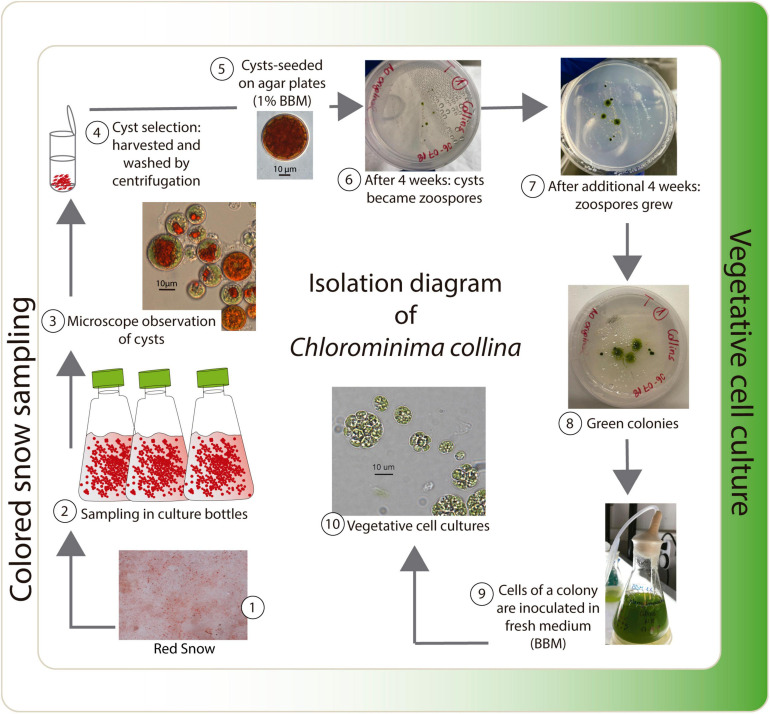
Isolation diagram of *Chlorominima collina* CCAP 6/1.

Red snow samples were collected from the surface of the Collins Glacier, King George Island, South Shetland Islands (62°10′5.412″S, 58°51′18.216″W), in February 2018. Each sample (approximately 400 g) was excavated 5 cm deep with a sterile metal spatula, transferred to an 18 cm × 20 cm sterile plastic bag and transported to the laboratory in the station “Base Profesor Julio Escudero.” After melting at 4°C, aliquots of 40 ml were stored in culture bottles (TR6000, TrueLine, United States) with 10 ml of 1% Bold’s basal medium (BBM, Bischoff and Bold, 1963). These bottles were transported in a cooling box at no more than 10°C to the Photobiology Laboratory of the Universidad Austral de Chile, in Valdivia, where they were kept in a cold room at 1°C. Samples were taken from one of the bottles in a 2.0-ml graduated microcentrifuge tube (Sorenson^TM^ BioScience, Inc., United States) under a laminar flow hood. Cysts were harvested and washed by centrifugation (3,500 rpm for 5 min at 4°C) four times with 1% BBM. Before cyst selection, the samples were examined under light microscopy to ensure that the cysts were free of other algae or microorganisms. Most of the observed cysts were in an early developing stage. Individual cysts were isolated manually under a microscope with a sterile glass micropipette (from Pasteur pipette) and then sown on agar plates with 1% BBM. The plates were kept in a cold room at 2°C with a photoperiod of 16:8 h light:dark (L:D) at photosynthetically active radiation (PAR) of 20–30 μmol m^–2^ s^–1^ provided by fluorescent tubes (T8, 36W-cold white, Westinghouse). The first green colonies were obtained after 8 weeks (Figure 1). A strain was isolated by means of serial dilutions in combination with differential centrifugation (Andersen, 2005). The isolated strain was transferred to Erlenmeyer flasks containing 1% BBM and cultivated in a cold room at 2°C. The isolate has been deposited and is available in the Culture Collection of Algae and Protozoa (CCAP), SAMS Limited, Scottish Marine institute, under strain number CCAP 6/1.

### Light and Electron Microscopy

The isolate was studied under light microscopy with an Olympus BX51 (Tokyo, Japan). Photomicrographs were taken with a QImaging MicroPublisher 5.0 digital camera, with Real Time Viewing (RTV). The QCapture Pro-6.0 (Teledyne Photometrics, Tucson, AR, United States) microimaging software was used to process images and obtain morphometric measurements of the cells. For transmission electron microscopy (TEM) two samples were taken, one of the mother culture with fresh medium and other from an old culture, both kept at 2°C. These samples were fixed in 2.5% glutaraldehyde and 0.1 M sodium cacodylate buffer (pH 7.2) for 24 h. Samples were washed with the cacodylate buffer (3 × 15 min) and subsequently fixed in 1% OsO_4_ at 4°C under shaking. After washing with deionized water (3 × 15 min) the fixed samples were dehydrated through a series of ethanol (35, 50, 70, 80, 96, and 100% for 10 min each), transferred to acetone (3 × 100% for 15 min) and finally embedded in epon resin: acetone-epon (1:1) from the EMBed-812 kit and then left in neat epon for polymerization at 60 °C during 24 h. Semi-fine sections (stained with toluidine blue) and ultra-fine sections were prepared with a Leica EM UC7 automatic ultramicrotome (Leica, Germany) and stained with 2% aqueous uranyl acetate and lead citrate for 5 min. The sections were examined using a Libra 120 Plus Transmission Electron Microscope, 80,000 KV acceleration voltage (Carl Zeiss, Germany). Photomicrographs were obtained using a Veleta CCD camera (EMSIS) equipped with Zemas V2.0 image analysis software. The images were tagged with Adobe Illustrator 2019 (version 23.03).

### DNA Extraction, PCR and Sequencing

Total genomic DNA was extracted with the GenElute Plant Genomic DNA Miniprep Kit (Sigma-Aldrich). According to this protocol, cells were ground in a mortar and pestle with liquid nitrogen, until obtaining 100 mg of powder. PCRs were performed using KAPA Taq HotStart PCR Kit, in a total volume of 12.5 μl (6.25 μl of the mix, 2.5 μl of primers and 1.25 μl of DNA). The 18S rRNA gene was amplified using the universal eukaryotic primers NS1 (White et al., 1990) and 18L (Hamby et al., 1988), with the cycling program as described in a previous study (Barcytė et al., 2018a). The region was sequenced with primers NS1 (White et al., 1990), 891F, 1122F (T. Friedl, unpublished) 18L (Hamby et al., 1988), 895R (Remias et al., 2012), and 1122R (T Friedl, unpublished). The complete region of the ITS was amplified with the ITS1 and ITS4 primers (White et al., 1990), following the cycling program of Barcytė et al. (2018a). The region was sequenced with primers ITS1 (White et al., 1990), 1800F (Friedl, 1996), 5.8 SbF (Mikhailyuk et al., 2008) and ITS2, ITS4, and LR3 (Vilgalys and Hester, 1990). Finally, a part of the ribulose-1,5-biphosphate carboxylase oxygenase (Rubisco) large subunit (rbcL) gene was amplified and sequenced with the primers rbcL1F and rbcL23R and the cycling program detailed in a previous study (Hoham et al., 2002). The PCR products were purified using the E.Z.N.A gel extraction Kit (Omega Bio-tek) and sequenced at Austral-Omics core facility at Universidad Austral de Chile. New sequences are available in GenBank under accession numbers MW554521 and MW553075.

### Phylogenetic Analysis

The BLAST algorithm (Altschul et al., 1990) from Geneious Prime (2019.2.1)^1^, was used to search for sequence identifiers of the 18S rRNA genes, rbcL and ITS of species closely related to the isolated strain, in addition to other representatives of the order Chlamydomonadales used in previous studies (Hoham et al., 2002; Barcytė et al., 2018a). Sequence assembly, alignment and verification to detect possible misaligned positions were made in Geneious Prime (2019.2.1). The 18S rRNA alignment comprised 136 OTUs (operational taxonomic units)/1,758 positions, while rbcL included 77 OTUs/872 positions. To perform the phylogenies, in both markers, the substitution parameter-rich model, GTR + I + G was used (Abadi et al., 2019). The maximum-likelihood phylogenies were performed in IQ-TREE Web Server (Trifinopoulos et al., 2016) considering statistical support values of ultrafast bootstrapping (10,000 replicates). Additional support values based on Bayesian posterior probabilities were obtained in MrBayes 3.2.7a x 64 (Ronquist et al., 2012) with sequence data set divided by codon positions. Two runs of the Markov Monte Carlo chain (MCMC) were carried out for twenty million generations each with one single cold chain and three heated chains being used in the GTR + I + G evolutionary model. The trees were sampled every 100 generations. After 10^6^ generations the mean standard deviation of the divided frequencies fell below 0.006 and the potential downscaling factor (PSRF) approached 1,000–1,001 for the diagnostic convergence parameters. The final trees of IQ-TREE and MrBayes were observed in the FigTree v1.4.4 (Rambaut and Drummond, 2018).

### Secondary Structure Analysis of ITS2 rDNA

The annotation of the secondary structure of the ITS2 rDNA nuclear region (including the 5.8 and 28S flanking regions) of the new strain and only a few sequences of ITS2 of strains closely related obtained by BLAST algorithm (Altschul et al., 1990) was performed through the web server^2^ based on hidden Markov models (Keller et al., 2009), in conjunction with the ITS2 database (Schultz et al., 2006; Selig et al., 2008; Koetschan et al., 2010, 2012). Secondary structure of the annotated ITS2 sequences was predicted in RNAfold WebServer^3^ considering the minimum energy secondary structure and centroid secondary structure model of ITS2 (Hofacker, 2003) and then visualized by VARNA 3.93 (Darty et al., 2009). Finally, a sequences-structures alignment, including the five very close sequences [*Chloromonas* sp. CCCryo257-06 (HQ404888), *Chloromonas* sp. CCCryo244-06 (HQ404887), *Chloromonas* sp. CCCryo273-06 (HQ404890); *Chloromonas perforata* CCAP 11/43 (FR865585) and *Chlamydomonas applanata* CCAP 11/9 (FR865616)], was built with the ClustalW2 1.83 algorithm from 4SALE 1.7.1 (Seibel et al., 2006, 2008). This program was also used to detect compensatory base changes (CBCs) between the sequences-structures (Wolf et al., 2013). The alignment obtained in 4SALE was exported to build a neighbor-joining tree (Saitou and Nei, 1987) in MEGA X (Kumar et al., 2018). The evolutionary distances were computed using the Jukes-Cantor method (Jukes and Cantor, 1969). A matrix plot of CBCs was performed in PAST 4.03 software (Hammer et al., 2001).

### Determination of Thermal Thresholds

To confirm the psychrophilic or psychrotrophic characteristics of the new strain, an experiment considering three temperatures (2, 10, and 20°C) was performed. After counting in a Neubauer chamber, suspensions of 400,000 cells/mL (2 ml) of culture were inoculated in 24-well cell culture plates (Thermo Fisher Scientific, United States). To avoid evaporation and contamination, the microplates were sealed with parafilm. The microplates (24 replicates) were left for 12 h at their control temperature (2°C). Subsequently they were transferred to acclimatization chambers for 19 days, under illumination conditions of 20–30 μmol m^–2^ s^–1^ and 16:8 h L:D regime. Growth was measured as changes in *in vivo* relative fluorescence units (RFU) in a microplate reader (Varioskan Flash, Thermo Fisher Scientific, United States). Fluorescence was measured at excitation (λex = 435 nm) and emission (λem = 685 nm) wavelengths. The specific growth rate (μ) was calculated using the equation:

$$\mu =l\,n\,\left(N_{2}/N_{1}\right)/\left(t_{2}-\,t_{1}\right)$$

where N_1_ and N_2_ are the growth (RFU) at time 1 (*t*_1_) and time 2 (*t*_2_), respectively.

The maximum quantum yield of chlorophyll fluorescence of dark-adapted photosystem II (F_*v*_/F_*m*_) was measured with an Imaging-PAM fluorometer (Walz, Effeltrich, Germany). The saturation pulse method was used to determine the basal (F_0_) and maximum (F_*m*_) fluorescence performance of dark-adapted samples (10 min is sufficient in dense microalgal mass cultures). Simultaneously, samples (100 μL) were taken for the qualitative detection of reactive oxygen species (ROS) on the third day of treatment when a decrease in F_*v*_/F_*m*_ values was observed in the treatment at 20°C, indicating stress or photoinhibition. To compare ROS production, samples were examined at 2°C (control) and at 10°C. The *in vivo* production of ROS was examined using the CellROX^®^ Green fluorogenic probe (Thermo Fisher Scientific, United States) according to a previously described protocol (Cornejo-Corona et al., 2016). For the visualization of intracellular ROS, the fluorescence was observed in an Olympus BX51 epifluorescent microscope (Olympus Corporation, Tokyo, Japan). Photographs were taken with a QImaging MicroPublisher 5.0 digital camera and the QCapture Pro-6.0 microimaging software was used for image processing.

### Statistical Analysis

To examine how temperature and time influence RFU and F_*v*_/F_*m*_ signals, generalized linear mixed models (GLMM) (Breslow and Clayton, 1993) were performed. Temperature was considered in the analysis as a fixed factor with three levels (2, 10, and 20°C, considering 2°C as a control). Time was also considered as a fixed factor. In all the models, ID of individual microalgae was incorporated as a random effect. The models that examine F_*v*_/F_*m*_ were based on Beta distribution, using “logit” as the link function. In the case of the models that evaluate growth by *in vivo* fluorescence (RFU), a Gamma distribution was considered, using “log” as the link function. All the statistical modeling was carried out in the R program version 3.5.2 (R Core Team, 2018), using the glmmTMB (Brooks et al., 2017) and lm4 (Bates et al., 2015) packages. Model comparisons were made using Akaike Information Criterion (AIC) applied within the bbmle (Bolker and Team, 2017) and MuMIn (Barton, 2019) packages. To examine how much variation is explained by the models (*R*^2^ coefficients), functions of the performance packages (Lüdecke et al., 2020) and MuMIn (Barton, 2019) were used. The assumptions of the fitted models were evaluated with DHARMa package (Hartig, 2020). Subsequently, a *post hoc* Tukey test was performed on the best models fitted with the multcomp package (Hothorn et al., 2008) and emmeans (Lenth, 2019). Multiple comparisons were applied to analyze differences between the temperature treatments, as a function of the mean time. Significance was examined at the 5% level.

### RNA Isolation, Library Preparation and Sequencing

Samples of the isolated strain cultured at 2°C (total 30 ml) were centrifugated at 4,500 × *g* for 10 min at 4°C. The cell pellets were immediately frozen in liquid nitrogen and subsequently stored at −80°C until further RNA extraction. The total RNA of the frozen cells was extracted and purified from 100 mg of cell powder, according to the protocols of the SpectrumTM Plant Total RNA kit (Sigma-Aldrich). The RNA concentration was quantified by Qubit 3.0 Fluorometric Quantification (Thermo Fisher Scientific) and its integrity (RIN = 9.8) evaluated by Fragment Analyzer—Advanced Analytical Technologies, Inc., (AATI) and Agilent DNF-471RNA kit. The library was prepared taking 4 μg of RNA sample. To isolate the mRNA, molecules containing poly-A, poly-T-oligo attached magnetic beads (Illumina) were used. Then the mRNA was purified and fragmented (200–700 nt) by divalent cations at 94 °C for 5 min. The purified mRNA was used for the construction of the cDNA library using the KAPA Stranded RNA-Seq Library Preparation Kit Illumina^®^ Platforms. Fragment analyzer system was used to evaluate the quality of the library. The libraries obtained were sequenced in Illumina Nextseq550 at the ChileGenómico research center (Facultad de Medicina, Universidad de Chile). After sequencing, the output was transformed by base-calling into sequences, which is the “raw reads” output in fastq format. All reads have been uploaded in the Sequence Read Archive (SRA) at NCBI, under accession numbers PRJNA698241 and BioProject: SAMN17709520.

### Analysis, *de novo* Transcriptome Assembly and Annotation

The raw reads were filtered to eliminate low quality regions (Quality score ≥ 30), in the prinseq-lite software (Schmieder and Edwards, 2011). Thus, pair-end reads with primer or adapter sequences and reads with more than 10% of the bases under the established quality were removed. After the read cleaning, *de novo* assembly was performed using the Trinity program (Grabherr et al., 2011). The functional annotation of the assembled sequences was performed using BLAST algorithm (*E*-value of < 10-5) against the databases: UniProt (UniProt Consortium, 2019), GO (Ashburner et al., 2000), and NCBI non-redundant protein database (NR) (Deng et al., 2006). To generate the gene ontology (GO) assignments, the Blast2GO program was applied (Conesa et al., 2005; Götz et al., 2008).

## Results

### Morphological and Ultrastructural Features

**FIGURE 2:**
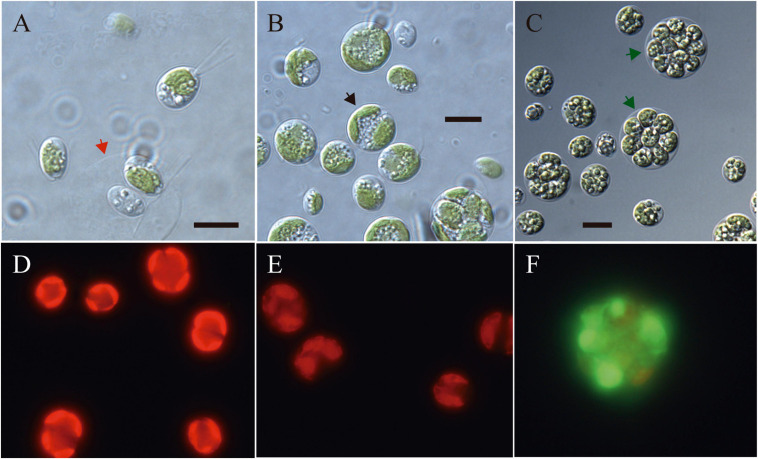
**(A–C)** Light microscopy images of morphology of *Chlorominima collina* CCAP 6/1 **(A)** Zoospores with two flagella of equal length. The red arrow indicates the maternal cell wall, from which the zoospores emerged. **(B)** Vegetative cells in the process of cell division. Black arrow points to a cell in division. **(C)** Mature cells with higher amounts of starch grains and lipid droplets. Green arrows indicate zoospores within mother cell wall. **(D–F)** Fluorescence images of *Chlorominima collina* using CellROX Green dye for detection of ROS *in vivo* on the third day of exposure at the control temperature of 2°C **(D)** and at the treatment temperatures of 10°C **(E)** and 20°C **(F)**. In panels **(D,E)** red stained chloroplasts of dividing cells are observed and in panel **(F)** the dividing cell (≥4 zoospores) is almost completely stained green. Scale bars: 10 μm.

**FIGURE 3:**
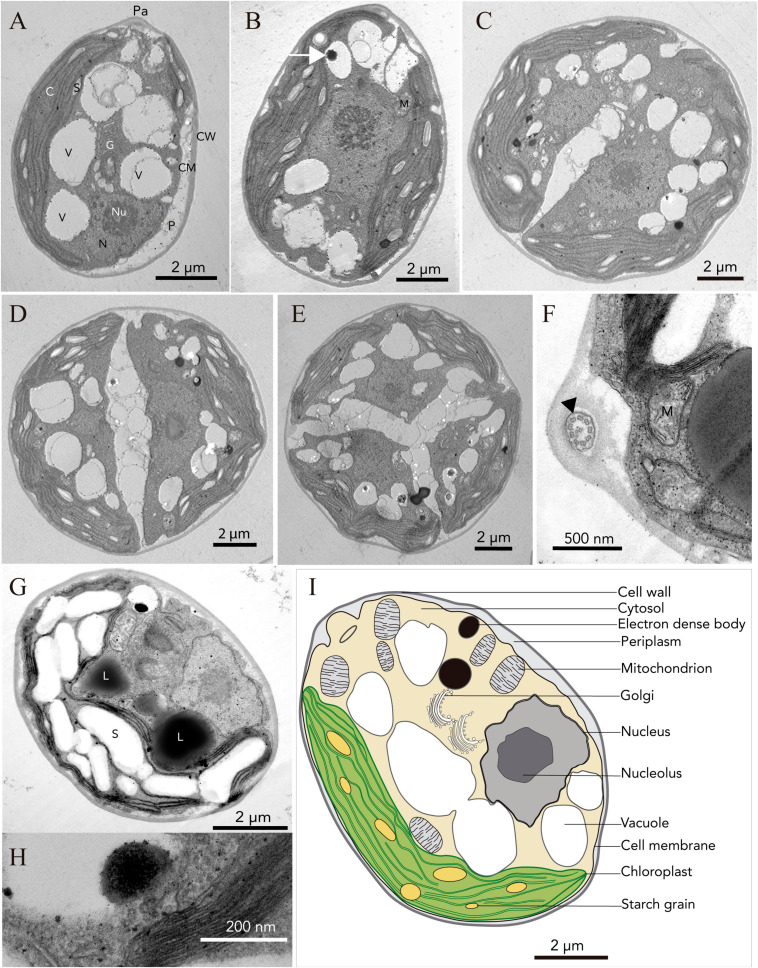
Ultrastructure (TEM) of *Chlorominima collina* CCAP 6/1 **(A)** Longitudinal section of a zoospore. **(B)** Mature vegetative cell (white arrow: electron dense body). **(C–E)** Zoospores in the process of cell division, where the protoplast is divided transversely. **(F)** Cross section of the flagellum showing the 9 + 2 axonema structure (black arrow). **(G)** Old culture cell. Pa, papilla; C, chloroplast; S, starch grains; V, vacuole; N, nucleus; Nu, nucleolus; G, Golgi apparatus; P, periplasm; CM, cell membrane; CW, cell wall; M, mitochondria; L, lipid droplet. **(H)** Plastoglobule in the chloroplast. **(I)** Consensus schematic drawing of a vegetative cell of *Cm. collina*.

Light microscopy revealed the presence of vegetative cells of ellipsoidal or ellipsoidal-cylindrical shape with rounded or even spherical posterior end, of 9–11 μm long and 6–12 μm width. Figure 2, with a smooth but noticeable cell wall and a discrete, hemispherical papilla (Figure 3). Two flagella, located apically, of equal length, 1.0 × cell length or longer, emerged under the papilla. TEM micrographs corroborate the presence of flagella with a cross section showing the axonema (Figure 3F). The young cells presented a single parietal chloroplast (dorsal side of the cell), laminated, shaped like a C, which occupies a large part of the cell volume. Eyespots were not observed in all stages of the vegetative phase, nor pyrenoids, but small grains of starch were distinguished, which were dispersed between the intertylakoidal spaces, along the chloroplast (Figure 3A). The size of the starch grains increases in cells from old cultures (Figure 3G). In mature cells it is possible to note how the chloroplast partially surrounds the nucleus (Figure 3B) until cell division occurs. Therefore, this alga reproduces asexually by forming two (Figures 3C–E) to eight motile zoospores (sometimes ten zoospores can be formed, even up 16). Motility was observed when the aliquots of the mother culture were transferred to a fresh medium, similar as has been reported for *Chloromonas arctica* (Barcytė et al., 2018a). However, unlike *Chloromonas arctica*, in *Chlorominima collina* motility was only maintained at low temperatures. A single nucleus was observed, which was located in the posterior region of the cell, eccentric, rather lateral (Figure 3A). In addition, in young cells, a wide periplasmic space was noted. Several cytoplasmatic globular vacuoles were observed, which can be empty or containing electron-dense deposits. Large oil droplets were evident only in cells from old cultures (Figure 3G). In these cells, plastoglobuli were also noted in the chloroplast (Figure 3H).

### Molecular Phylogeny and Secondary Structure Analysis of ITS2

**FIGURE 4:**
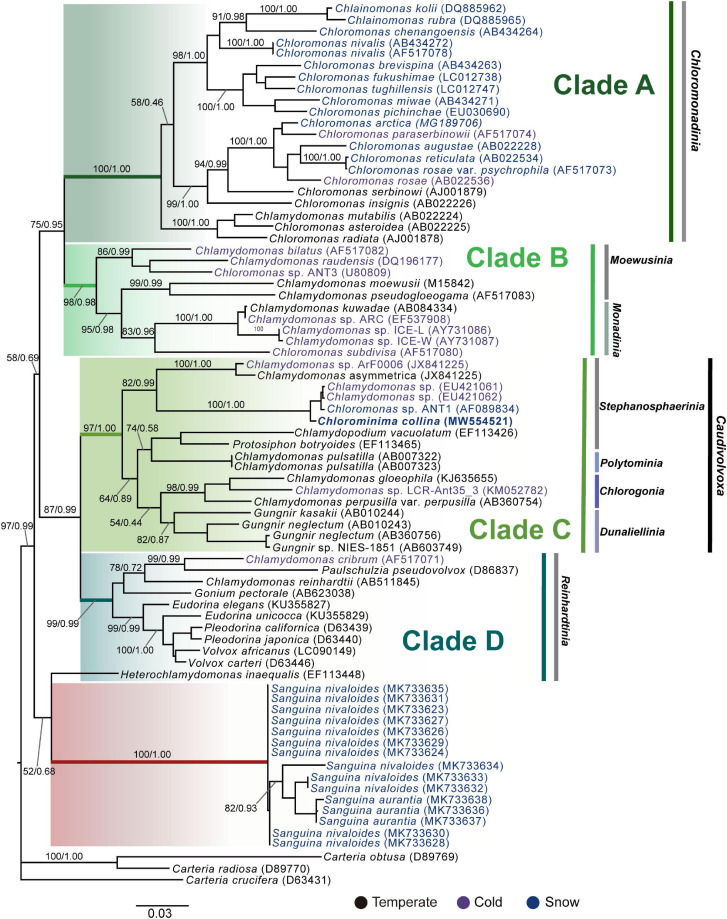
Maximum-likelihood phylogram of rbcL gene sequences. The position of *Chlorominima collina* CCAP 6/1 and other related members of the order Chlamydomonadales. Clades A, B, C, and D were delimited according to Hoham et al. (2002). The names of the clades were designated according to Nakada et al. (2008). The new genus of *Sanguina* snow algae is specified (Procházková et al., 2019a). Representatives of the genus *Carteria* were used as outgroup. The numbers on the branches indicate statistical support values (Maximum-likelihood bootstraps/Bayesian posterior probabilities). Snow taxa are marked in blue, taxa from other cold environments, not from snow, are marked in purple and the rest of the taxa from other temperate environments are written in black. The lower bar indicates changes by nucleotide position (0.03).

**FIGURE 5:**
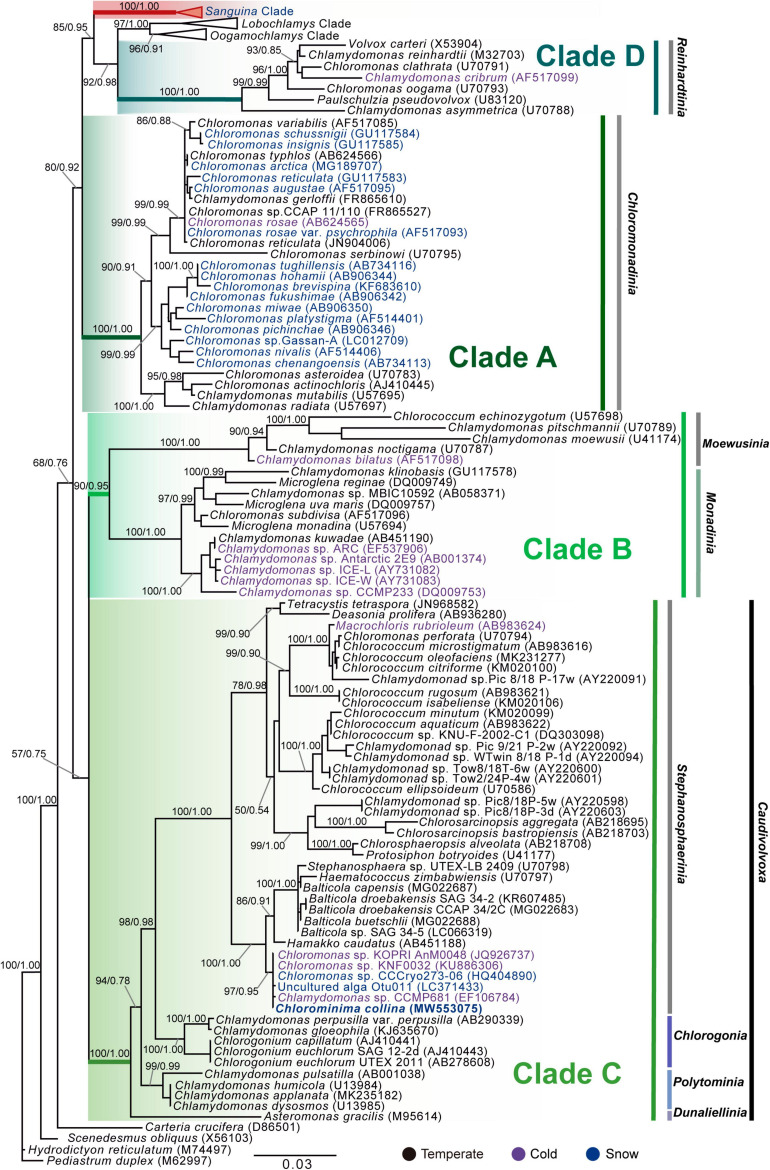
Maximum-likelihood phylogram of the sequences of the 18S rRNA gene of *Chlorominima collina* CCAP 6/1 and other representative members of the order Chlamydomonadales. Clades A, B, C, and D were circumscribed according to Buchheim et al. (1997) and Hoham et al. (2002). The new genus of *Sanguina* snow algae is specified (Procházková et al., 2019a). *Carteria crucifera*, *Tetradesmus obliquus*, *Hydrodictyon reticulatum*, and *Pediastrum duplex* were considered as outgroup. The names of the clades were designated according to Nakada et al. (2008). The numbers on the branches indicate statistical support values (Maximum-likelihood bootstraps/Bayesian posterior probabilities). Snow taxa are marked in blue, taxa from other cold environments, not from snow, are marked in purple and the rest of the taxa from other temperate environments are written in black. The lower bar indicates changes by nucleotide position (0.03).

**FIGURE 6:**
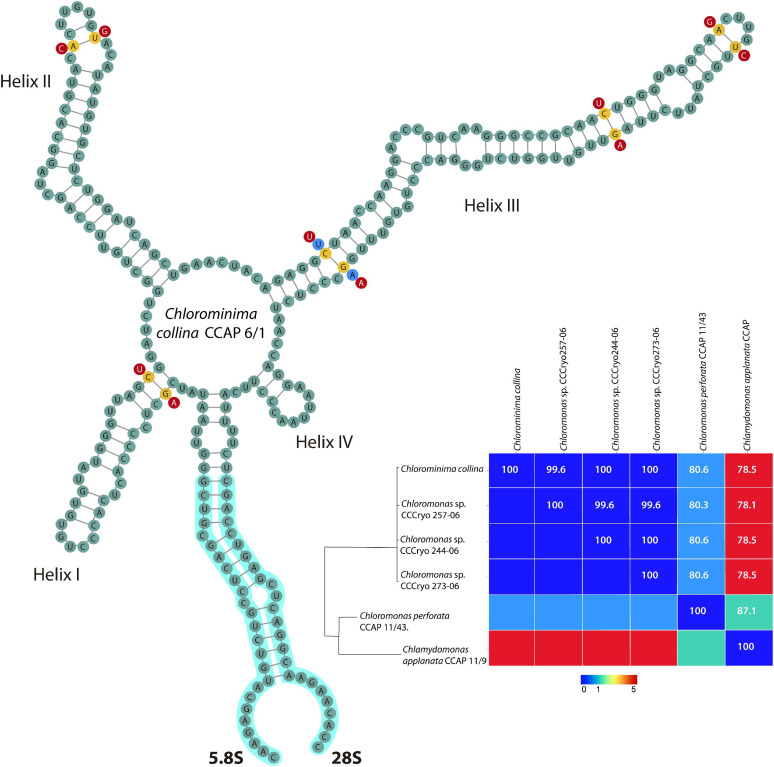
Secondary structure model of the ITS2 rDNA of *Chlorominima collina* CCAP 6/1. The positions of CBC are shown in yellow circles between *Chlorominima collina* and *Chloromonas perforata* CCAP 11/43 (light blue circles) and *Chlamydomonas applanata* CCAP 11/9 (red circles). The Matrix under the structure shows compensatory base changes (CBCs) by color under diagonal and identity percent (above diagonal) among the respective strains considered for the analysis of the secondary structure of ITS2.

*Chlorominima (Cm.) collina* was located within clade C, one of the four main lineages identified for the *Chloromonas (Cr.)* and *Chlamydomonas (Cd.)* complex (Buchheim et al., 1997; Hoham et al., 2002). Specifically, *Cm. collina* is assigned within the *Stephanosphaerinia* phylogroup, which is one of the 21 monophyletic groups recognized for the class Chlorophyceae, order Chlamydomonadales (Nakada et al., 2008). In the phylogeny of the rbcL gene (Figure 4) the new strain is located with high support [Maximum likelihood support (ML): 100/Bayesian inference (BI): 1.00] within clade C, in a subclade that contains unidentified polar strains assigned to the genus *Chloromonas* and *Chlamydomonas*. In this subclade, *Cm. collina* is more similar to the rbcL gene sequences of Genbank of *Chloromonas* sp. ANT1 (AF089834) and *Chlamydomonas* sp. CCMP681 (EU421062 and EU421061) of which it differs by 11 nucleotides. Compared with other similar rbcL gene sequences, *Cm. collina* differs by 90 and 98 nucleotides from *Chlamydomonas gloeophila* UTEX_607 (KJ635655) and *Chlamydomonas pulsatilla* CCAP11-106 (AB007322), respectively. Phylogenetic analyses based on 18S rRNA gene corroborated the assignment of *Cm. collina* in the *Stephanosphaerinia* phylogroup (Figure 5), where it also forms a subclade highly supported (ML: 100/BI: 1.00), with other unidentified polar strains assigned to *Chloromonas* and *Chlamydomonas*. *Cm. collina* presents 100% identity to the 18S rRNA gene sequence of *Chloromonas* sp. KNF0032 (KU886306) and differs by two nucleotides from *Chloromonas* sp. CCCryo273-06 (HQ404890) and from *Chlamydomonas* sp. CCMP681 (EF106784). The secondary structure analyses of the ITS2 rDNA revealed a high similarity between *Cm. collina* and strains of *Chloromonas* sp. (CCCryo273-06, CCCryo244-06, and CCCryo257-06) with identity of 99.6–100%. These sequences did not show CBCs (Figure 6). Other strains with similar ITS2 sequences were *Chloromonas perforata* CCAP 11/43 and *Chlamydomonas applanata* CCAP 11/9, which are representatives of *Caudivolvoxa* and differ from *Cm. collina* by 1–5 CBCs, respectively, presenting an identity percent of 80.6 and 78.5, respectively (Figure 6).

### Thermal Responses of *Chlorominima collina*

**FIGURE 7:**
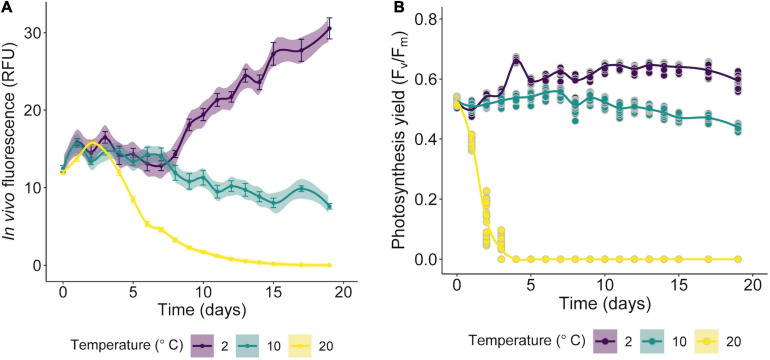
Growth curves of *Chlorominima collina* CCAP 6/1 based on RFU values **(A)** and efficiency of the quantum yield of photosystem II (PSII) (F_*v*_/F_*m*_) **(B)** exposed to 2, 10, and 20°C. The lines show the trends, and the shading indicates the confidence intervals (CI). In the RFU graphs, each point is the mean of 24 replicates and the bars represent the standard error (SE). For F_*v*_/F_*m*_ data, each point is an average of 24 replicates (raw data).

The isolated alga tolerates only temperatures < 10°C (Figure 7), while at 20°C declines in growth (based RFU values) and in the specific growth rates (Supplementary Figure 1) were observed. Preliminary tests indicated that the optimum growth temperature for *Cm. collina* is around 4°C (results not shown). Low F_*v*_/F_*m*_ values indicated that temperatures of 20°C in interaction with time were stressful to photochemistry of *Cm. collina* (glmmTMB; *p* < 0.001; Supplementary Table 1 and Figure 7B). In fact, after 3 days at 20°C, F_*v*_/F_*m*_ reached values close to 0.1, which was accompanied by the *in vivo* detection of ROS (Figure 2F). This caused a significant decrease in growth (RFU) at 20°C over time (glmer; *p* < 0.001; Supplementary Table 1 and Figure 7A). Conversely, *Cm. collina* cells kept under control temperature (2°C) showed active growth (Figure 7A) and high levels of photochemical activity (F_*v*_/F_*m*_ values ≤ 0.6; Figure 7B), while *in vivo* ROS was not detected (Figure 2D). Interestingly, the new strain maintains relatively constant F_*v*_/F_*m*_ and growth values during the first days at 10°C (Figure 7) and ROS formation was not evident on the third day at this temperature (Figure 2E). After this period, a decline in F_*v*_/F_*m*_ and growth was observed, suggesting that 10°C is a thermal tolerance limit for *Cm. collina*.

### *De novo* Transcriptome Assembly and Annotation

**FIGURE 8:**
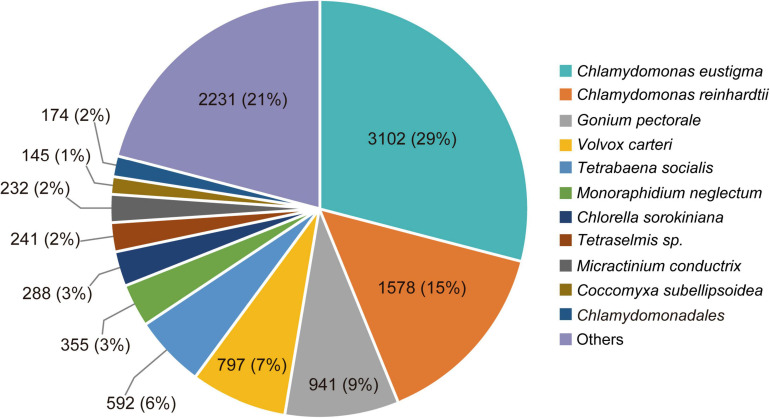
Species distribution of the top BLAST hits by NR database annotation for *Chlorominima collina* CCAP 6/1. Percentage distribution of the 10 most successful species identified for each BLAST-assembled sequence against the NCBI non-redundant protein database. In addition to the percentage value, the number of genes noted per species is indicated.

**FIGURE 9:**
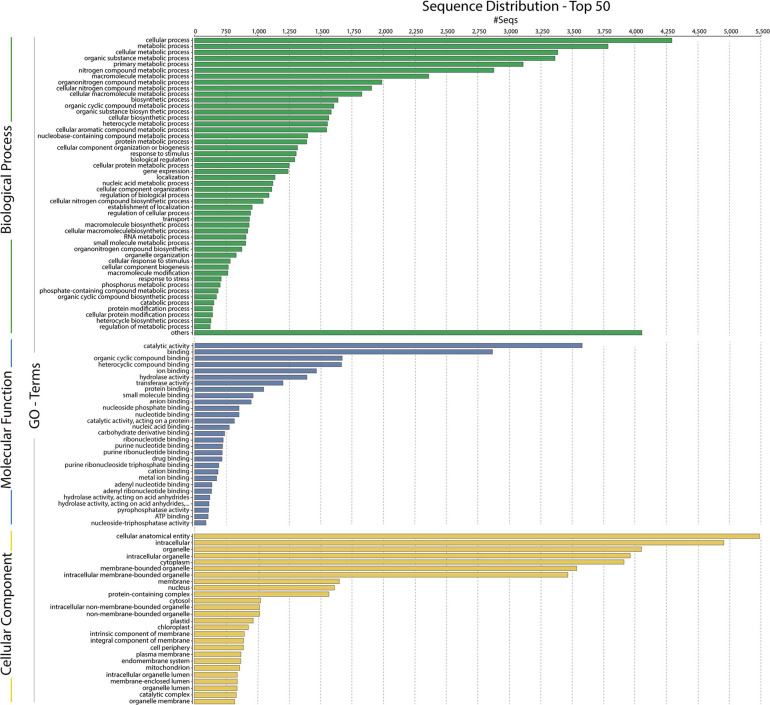
Gene ontology (GO) assignments for the *Chlorominima collina* CCAP 6/1 transcriptome. Assignments generated in Blast2GO predict the participation of genes in biological processes (green); molecular functions (blue) and cellular components (yellow).

In total, 2,670,333 crude Ilumina PE readings were obtained. Following removal of low-quality readings and with adapters, 1,539,352 readings were obtained. These RNA-seq reads were subjected to a *de novo* transcriptome assembly, which yielded 37,401 transcripts with N50 of 881 bp and an average length of 643.88 bp (Table 1). A number of annotated sequences (10,676) had a significant hit identified by BLAST search against Uniprot and NCBI, 79% of these hits were assigned to sequences of green algae (Chlorophyta), mainly members of the Chlamydomonadales order, while the rest corresponded to other divisions of algae (Figure 8). From the annotations against the GO database, it was possible to infer the presence of genes in the categories of GO (Figure 9). In the category “biological process” the top GO terms were “cellular process” (GO: 0009987), “metabolic process” (GO: 0008152), and “cellular metabolic process” (GO: 0044237). For “molecular function” the top GO terms were “catalytic activity” (GO: 0003824) and “binding” (GO: 0005488). In the “cellular component” category, the GO terms “cellular anatomical entity” (GO: 0110165) and “intracellular” (GO: 0005622) were the most enriched (Figure 9). Transcripts annotated against the Uniprot database using BLAST similarity, indicated well-known stress-responsive genes (Table 2), e.g., genes encoding “ABC transporters” or “calcium/calmodulin-dependent protein kinase” and “heat shock proteins.” The identification of several genes related to photosynthesis such as “oxygen-evolving enhancer proteins” (e.g., TRINITY_DN6396), or “Rubisco” (TRINITY_DN13299), together with other genes involved in the translation process such as “elongation factors” (e.g., TRINITY_DN21381) and “ribosomal proteins” (e.g., TRINITY_DN10395) indicate an active metabolism of *Cm. collina* at 2°C. At least 12 transcripts (Table 3) encoding antifreeze glycoprotein and ice-binding proteins were observed. A large number of transcripts associated with biosynthesis of fatty acids, especially polyunsaturated fatty acids (PUFAs), triacylglycerol (TAG), secondary carotenoids were also identified (Table 4). Additionally, genes involved in synthesis of betaine were identified (Table 5).

**TABLE 1:** Transcriptome sequencing and summary statistics of *de novo* assembly.

**Number/length**	
Number of reads from Nextseq550 (2 × 150 pb)	2,670,333
High-quality (HQ) reads	1,539.352
Total trinity transcripts	37,401
Total trinity genes	33,337
Percent GC	54,82
Average contig length	643,88 bp
N50	881

**TABLE 2:** Stress-responsive genes present in the *Chlorominima collina* trascriptome.

***De novo* assembled sequence ID**	**GO molecular function (species)**
TRINITY_DN1216	ABC transporter F family member 5 (*Tetrabaena socialis*)
TRINITY_DN25081	ABC transporter B family member 25 *(Anthurium amnicola*)
TRINITY_DN19387	Leucine-rich repeat-containing protein 1 *(Tetrabaena socialis*)
TRINITY_DN747	Leucine-rich repeat-containing protein 40 (*Auxenochlorella protothecoides*)
TRINITY_DN7218	Hypersensitive-induced response protein 3 (*Tetrabaena socialis*)
TRINITY_DN6510	Calcium/calmodulin-dependent protein kinase 1 Da (*Lepisosteus oculatus)*
TRINITY_DN10034	Heat shock factor 1 (*Chlamydomonas reinhardtii*)
TRINITY_DN4825	Activator heat shock protein ATPase (*Monoraphidium neglectum*)
TRINITY_DN9027	Heat shock cognate 71 kDa protein-like (*Saccoglossus kowalevskii*)
TRINITY_DN10375	Heat shock protein 70a (*Dunaliella salina*)
TRINITY_DN10700	Heat shock protein 70D (*Chlamydomonas reinhardtii*)

**TABLE 3:** Putative Antifreeze and Ice-Binding proteins found in transcriptome of *Chlorominima collina.*

***De novo* assembled sequence ID**	**GO molecular function (species)**
TRINITY_DN12789	Antifreeze glycoprotein (*Rhodosporidium toruloides*)
TRINITY_DN1603	Ice-binding protein-2 (*Chlamydomonas* sp. CCMP681)
TRINITY_DN1624	Ice-binding protein-4 (*Chlamydomonas* sp. CCMP681)
TRINITY_DN16614	Ice-binding protein-4 (*Chlamydomonas* sp. CCMP681)
TRINITY_DN25734	Ice-binding protein-4 (*Chlamydomonas* sp. CCMP681)
TRINITY_DN26347	Ice-binding protein-4 (*Chlamydomonas* sp. CCMP681)
TRINITY_DN27778	Ice-binding protein (*Chloromonas* sp.)
TRINITY_DN4443	Ice-binding protein-4 (*Chlamydomonas* sp. CCMP681)
TRINITY_DN6301	Ice-binding protein-4 (*Chlamydomonas* sp. CCMP681)
TRINITY_DN6423	Ice-binding protein-4 (*Chlamydomonas* sp. CCMP681)
TRINITY_DN7238	Ice-binding protein-3 (*Chlamydomonas* sp. CCMP681)
TRINITY_DN8708	Ice-binding protein (*Chloromonas* sp.)

**TABLE 4:** Putative desaturases and other enzymes involved in the biosynthesis of fatty acids, triacylglycerol and secondary carotenoids present in the transcriptome of *Chlorominima collina.*

***De novo* assembled sequence ID**	**GO molecular function (species)**
TRINITY_DN12058	Fatty acid elongase 1 (*Orychophragmus violaceus*)
TRINITY_DN12819	Fatty acid desaturase 4 (*Klebsormidium nitens*)
TRINITY_DN1377	Delta-12-fatty acid desaturase (*Glaciozyma antarctica*)
TRINITY_DN15049	Putative long-chain-alcohol O-fatty-acyltransferase 5 (*Chlorella sorokiniana*)
TRINITY_DN18272	Fatty acid synthase subunit alpha (*Rhodosporidium toruloides*)
TRINITY_DN22769	Cyclopropane-fatty-acyl-phospholipid synthase (*Tetrabaena socialis*)
TRINITY_DN27022	Elongation of fatty acids protein (*Rhodotorula graminis*)
TRINITY_DN30110	Putative long-chain-alcohol O-fatty-acyltransferase (*Tetrabaena socialis*)
TRINITY_DN6285	Fatty acid desaturase 2 (*Jatropha curcas*)
TRINITY_DN6913	Fatty acid desaturase 5 (*Theobroma cacao*)
TRINITY_DN6936	Fatty acid delta-6-desaturase (*Lobosphaera incisa*)
TRINITY_DN7069	Omega-3-fatty acid desaturase (*Chlamydomonas reinhardtii*)
TRINITY_DN6274	Stearoyl-ACP-desaturase (*Haematococcus lacustris*)
TRINITY_DN15482	Cytochrome P450, C-22 desaturase (*Chlamydomonas reinhardtii*)
TRINITY_DN9974	Triacylglycerol lipase-like protein (*Chlamydomonas reinhardtii*)
TRINITY_DN11143	Diacylglycerol kinase (*Chlamydomonas eustigma*)
TRINITY_DN18922	Putative phospholipid:diacylglycerol acyltransferase 2 (*Arabidopsis thaliana*)
TRINITY_DN19602	Diacylglycerol kinase (*Volvox carteri* f. *nagariensis*)
TRINITY_DN3878	Diacylglycerol acyltransferase type 2 (*Ettlia oleoabundans*)
TRINITY_DN6178	Diacylglycerol acyltransferase type 2 (*Chlamydomonas reinhardtii*)
TRINITY_DN6279	Glycerol-3-phosphate acyltransferase, chloroplastic (*Chlamydomonas eustigma*)
TRINITY_DN7159	1-acyl-sn-glycerol-3-phosphate acyltransferase (*Chlamydomonas reinhardtii*)
TRINITY_DN7140	Glycerol-3-phosphate dehydrogenase [NAD(+)] *(Chlamydomonas reinhardtii*)
TRINITY_DN239	Putative stearoyl-CoA 9-desaturase (*Leucosporidium creatinivorum*)
TRINITY_DN10651	Zeta-carotene desaturase (*Auxenochlorella protothecoides*)
TRINITY_DN19166	Zeta-carotene desaturase (*Haematococcus lacustris*)
TRINITY_DN2370	Phytoene desaturase (*Haematococcus lacustris*)

**TABLE 5:** Putative betaine aldehyde dehydrogenases genes present in the transcriptome of *Chlorominima collina*.

***De novo* assembled sequence ID**	**GO molecular function (species)**
TRINITY_DN13477	Betaine aldehyde dehydrogenase 2, mitochondrial (*Arabidopsis thaliana*)
TRINITY_DN21321	Betaine aldehyde dehydrogenase 2, mitochondrial (*Tarenaya hassleriana*)
TRINITY_DN5490	Betaine aldehyde dehydrogenase 2 (*Phoenix dactylifera*)

### Taxonomic Treatment

#### *Chlorominima* Gálvez gen nov.

Type species: *Chlorominima collina* Gálvez sp. nov.

Etymology: the name reflects the morphological similarity with the genus *Chloromonas*, and in turn refers to a small cell size.

Registration: http://phycobank.org/102721

Description: ellipsoidal cells with rounded posterior end, approximately 11 μm long and 12 width, with two flagella of equal length at the anterior end. A single parietal chloroplast on the dorsal side of the cell, without pyrenoids, eyespot absent, and starch grains scattered between intertylakoidal spaces. Nucleus single, positioned in the posterior region of the cell, eccentric. Smooth and noticeable cell wall. Discrete hemispheric papilla. More than five contractile vacuoles irregularly distributed on the protoplast surface. Asexual reproduction through the formation of 2–16 zoospores within the parental cell wall.

#### *Chlorominima collina* Gálvez sp. nov.

Registration: http://phycobank.org/102722

Description: vegetative cells, ellipsoidal, ellipsoidal-cylindrical or spherical with rounded posterior end, of 9–11 μm length and 6–12 μm width, biflagellate with flagella of equal length (1.0 × cell length or longer). Single chloroplast, laminate and parietal in the dorsal side of the cell, defined as C-shaped, with starch grains scattered between intertylakoidal spaces. Pyrenoid and eyespot absent. Nucleus single, positioned in the posterior region of cell, eccentric, rather lateral. Approximately 5–6 contractile vacuoles distributed irregularly on the protoplast. Discrete and hemispherical anterior papilla. Young cells present wide periplasmic space. Old cells lose their flagella, are globular in shape with cytoplasmic lipid droplets that occupy most of the cell volume and increase the size of starch grains. Asexual reproduction through the formation of generally up to eight zoospores within the cell wall. In old cultures, formations of up to 16 zoospores can also be observed within the cell wall. Sexual reproduction was not evident. Cell aggregates were not observed in cultures.

Holotype: the strain is preserved permanently at 2°C at the Photobiology Laboratory of the Universidad Austral de Chile, Valdivia, Chile. It has also been deposited in the CCAP, based in Scottish Association for Marine Science near Oban, Scotland, United Kingdom^4^, under strain number CCAP 6/1. Figures 2, 3 show the morphology of the holotype.

Type locality: red snow from the Collins Glacier, King George Island, South Shetland Islands (62°10′5.412″S, 58°51′18.216″W), Western Antarctic Peninsula.

Etymology: the species name was chosen to emphasize the holotype’s sampling area, the Collins Glacier.

Distribution: Antarctica.

## Discussion

Recent studies have demonstrated that snow algae are taxonomically diverse. In part, this has been possible due to the application of polyphasic approaches that have allowed the identification of new species (Matsuzaki et al., 2018, 2019; Procházková et al., 2019b), even a new genus, *Sanguina* has been recognized as a dominant component of the snow communities (Procházková et al., 2019a). This knowledge is crucial, since it allow building a basis for characterizing the colored snow blooms, allowing to describe the genetic diversity of snow algae and their dispersal (Segawa et al., 2018; Soto et al., 2020). Thus, the description of a new Antarctic genus, *Chlorominima* with the species type *Cm. collina*, together with the integration of molecular and physiological approaches serves not only to improve our knowledge about Antarctic taxonomic diversity, but also provides elements to understand its functional attributes, reflecting adaptations to the snow ecosystem.

New characterizations of snow communities developed in the Fildes peninsula, King George Island, have reported in the red snow a low abundance of *Chloromonas* sp. CCCryo273-06 (HQ404890) a integrant of the polar subclade of *Chlorominima* (Luo et al., 2020). This only confirms the presence of representatives of *Chlorominima* in the red snow, which probably coexist with members of *Sanguina* and *Chlainomonas* (Procházková et al., 2019a; Luo et al., 2020) but does not prove that the cells of *Chloromonas* sp. CCCryo273-06 or the isolated strain in this study have germinated from cysts, as a conclusive methodology it is suggested to perform single-cell sequencing, to verify the taxonomic identity of the cysts.

### Morphology and Ultrastructure of *Chlorominima collina*

The characteristics observed in the vegetative phase of our new strain were similar to the morphological traits described for snow or ice species of *Chloromonas* (Ettl, 1970, 1983), including the common absence of pyrenoids. This contrasts with the presence of pyrenoids in taxa phylogenetically related to *Cm. collina*, such as *Chlamydomonas perpusilla* var. *perpusilla* (Nakada and Nozaki, 2007), *Hamakko caudatus* (Nakada and Nozaki, 2009), or *Haematococcus zimbabwiensis* (Buchheim et al., 2013), confirming that this structural trait may be absent or present within the *Chloromonas-Chlamydomonas* complex and therefore could not be relevant for the natural history of these algae (Hoham et al., 2002; Matsuzaki et al., 2012). In addition, *Cm. collina* lacks an eyespot, suggesting that this structure is not an essential component of the photoreceptor apparatus (Morel-Laurens and Feinleib, 1983), which has also been reported for other snow algal strains such as *Cr. pichinchae* (Hoham, 1975a), *Cr. krienitzii* (Matsuzaki et al., 2015), or *Cr*. *fukushimae* (Matsuzaki et al., 2014). Similar to the latter species, *Cm. collina* has a parietal, laminate chloroplast but is defined as C-shaped, that is unusual among snow algal species of *Chloromonas* exhibiting a cup-shaped chloroplast (Ling and Seppelt, 1998; Matsuzaki et al., 2014, 2019). Species closely related to *Cm. collina* such as *Hamakko caudatus* (Nakada and Nozaki, 2009) also have a parietal chloroplast, it also has a similar cell width (6–10 μm) to *Cm. collina*, but *Hamakko caudatus* have spindle cell shape and can have 10 contractile vacuoles and *Cm. collina* only 6. Other close species such as *Cd. perpusilla* var. *perpusilla* only present two apical contractile vacuoles (Nakada and Nozaki, 2007).

Furthermore, cells of *Cm. collina* have an average length between 9 and 11 μm, while the width does not exceed 12 μm, resembling cells of *Cr. alpina* and *Cr. miwae*, but they differ in chloroplast and cell morphology (Matsuzaki et al., 2019). Rather, the ellipsoidal shape of the cells of *Cm. collina* are comparable to *Cr. hoshawii* cells (Matsuzaki et al., 2018). This species does not present a discernible papilla and can only form up to four zoospores (rarely eight) within the parental cell wall, while *Cm. collina* can form up to eight zoospores (rarely 16), a feature reported also in *Cr. nivalis* (Matsuzaki et al., 2018) and *Cr. fukushimae* (Matsuzaki et al., 2014). Similar to the latter species, *Cm. collina* does not form cellular aggregates in cultures.

Although *Cm. collina* displays several typical characteristics of the *Chloromonas*, it is distinguished from the species described for this genus and from phylogenetically close species such as *Hamakko caudatus* by showing an unusual position of the nucleus, a wide periplasmic space between the inner and outer cell membranes, the presence of 5–6 contractile vacuoles, the small cell size that did not exceed 12 μm in both length and width, and the absence of pyrenoid and eyespot.

### Phylogenetic Position of *Chlorominima collina* and Phylogeny of Cold Tolerant Chlamydomonadales

*Chlorominima collina* CCAP 6/1 is placed in the phylogroup *Stephanosphaerinia*, or clade C (Nakada et al., 2008). This is unexpected since most snow algae occur in clade A or *Chloromonadinia* (Hoham et al., 2002). Specifically, within clade C, *Cm. collina* forms an independent lineage, close to other polar strains unidentified, but assigned to *Chloromonas* and *Chlamydomonas* (Supplementary Table 2). With the rbcL gene, *Cm. collina* is sister to *Chloromonas* ANT1 and *Chlamydomonas* sp. CCM681, for which there are no morphological descriptions that allow comparisons, only their adaptations to cold environments have been characterized (Devos et al., 1998; Raymond et al., 2009). By using the 18S rRNA gene, *Cm. collina* exhibits high identity with *Chloromonas* sp. KNF0032, both strains have a small cell size and an ovoid cell shape but differ in the shape of their chloroplasts and by the apparent presence of pyrenoid in *Chloromonas* sp. KNF0032 (Jung et al., 2016b). The insufficient description of *Chloromonas* sp. KNF0032 limits further conclusions, but it must also be taken into account that the evolutionary highly conserved 18S rRNA gene does not provide sufficient resolution to discriminate between closely related species (Barcytė et al., 2018a; Remias et al., 2018; Lutz et al., 2019). When the taxonomic status of *Cm. collina* was verified under the CBC species concept (Coleman, 2000; Müller et al., 2007) we observe the absence of CBC among *Cm. collina* and other strains known as *Chloromonas* sp. (CCCryo273-06, CCCryo244-06, and CCCryo257-06), which could indicate with a probability of ∼ 0.76 that these algae belong to the same species (Coleman, 2000; Müller et al., 2007). This scenario may be quite feasible given the proximity between the sampling sites of these algae (Supplementary Table 2). Although, it should also be noted that even without CBC, the named strains may belong to different species with a probability of ∼ 0.24 (Caisová et al., 2013; Škaloud and Rindi, 2013; Procházková et al., 2018). Even in some cases it has been suggested that morphological changes precede the emergence of a CBC (Pawłowska et al., 2013). However, these strains have not been described at the species level, so there are no descriptions that allow morphological comparisons. On the other hand, the presence of CBCs was found in helix III, one with *Chloromonas perforata* CCAP 11/43 and five with *Chlamydomonas applanata* CCAP 11/9, revealing that *Cm. collina* is a distinct taxon within *Caudivolvoxa* with a probability of ∼ 0.93 (Müller et al., 2007; Wolf et al., 2013).

In the phylogenies of the 18S rRNA gene and the rbcL gene it was possible to observe the four previously described clades (A, B, C, and D) for cold-tolerant taxa of *Chlamydomonas* and *Chloromonas* complex (Buchheim et al., 1997; Hoham et al., 2002), which reaffirms that these genera have colonized cold habitats at least five times during their evolutionary history (Hoham et al., 2002; Remias et al., 2010; Hoham and Remias, 2020). Additionally, by including the recent clade of *Sanguina*, another origin in cold habitats is added (Procházková et al., 2019a). In *Stephanosphaerinia*, in addition to the polar subclade of *Cm. collina* and related strains, there are other cold-adapted members such as *Macrochloris rubrioleum* CCCryo 340b-08, suggesting that there is more than one origin of cold-adaptation within of this clade. In addition, the present phylogenies show that the representatives of both *Chlamydomonas* and *Chloromonas* are not closely related, confirming the reported polyphilia of both genera (Nakayama et al., 1996; Pröschold et al., 2001; Barcytė et al., 2018a). This can be the result of a combination of factors such as the use of symplesiomorphies, environmentally variable characters, evolutionary convergence of vegetative morphologies, or maybe the omission of ecological preferences that can drive sympatric differentiation (Nakayama et al., 1996; Pröschold et al., 2001; Malavasi et al., 2016). In fact, this last point has resolved the classification of genera attaining problematic morphologies such as *Coccomyxa* (Malavasi et al., 2016). Interestingly, in *Chloromonas*-*Chlamydomonas* complex, the habitat has been strongly correlated with the phylogenetic history of these genera irrespective of morphology (Buchheim et al., 1997; Hoham et al., 2002; Barcytė et al., 2018b). The discovery of polar subclades in the Chlamydomonadales (Demchenko et al., 2012; Procházková et al., 2019a), such as observed in the present study, support the idea that extreme environments have promoted the evolution and speciation in unicellular chlorophytes (Pollio et al., 2005; Fučíková et al., 2014; Malavasi et al., 2016).

Considering that *Cm. collina* (i) forms an independent lineage within *Stephanosphaerinia*, (ii) it is sister to strains assigned to *Chloromonas* or *Chlamydomonas*, but which are not monophyletic with the type species of *Chloromonas* (*Cr. reticulata*) or *Chlamydomonas* (*Cd. reinhardtii*), so it is not can maintain the assigned generic identity and must be transferred to other genera (Pröschold et al., 2001), we propose a new genus *Chlorominima*, describing the type species *Cm. collina*, with the aim of generating a baseline to identify the rest of the members of this polar monophyletic lineage, contributing to the biodiversity of Chlamydomonadales.

### The Psychrophilic Character of *Chlorominima collina*: A Typical Feature of True Snow Algae

Physiological experiments revealed that *Cm. collina* is a psychrophilic organism, therefore the increase in temperature to 20°C causes in this strain a marked decrease of the maximum quantum yield of the PSII and oxidative stress on the third day of exposure. These responses were also correlated with a decline in growth. Probably it was a direct result of thermal stress, which can affect key metabolic functions, disrupting cellular homeostasis and uncoupling physiological processes (Suzuki and Mittler, 2006; Barati et al., 2019). Likewise, the effects of ROS vary not only according to temperature, but also according to the duration of heat stress (Dunn et al., 2004), which in this case was in scale of days. The maintenance of growth and photosynthetic performance or F_*v*_/F_*m*_ during the first days of exposure to 10°C and the subsequent decline of these parameters may indicate that this temperature is a physiological limit for *Cm. collina* similar to that observed in other snow algae (Hoham, 1975b; Devos et al., 1998). In addition to growth, the psychrophilic character was evaluated through the maximum quantum yield of PSII (F_*v*_/F_*m*_), since this physiological parameter is sensitive to alterations in early photochemical reactions revealing stress (Maxwell and Johnson, 2000). These changes were observed in *Cm. collina* after 24 h of exposure at 20°C with a decrease in F_*V*_/F_*m*_ below 0.4 compared to F_*V*_/F_*m*_ above 0.5 at 2°C (Figure 7B). Such alterations may not be detected using growth alone, so evaluating growth alone is inappropriate to define a psychrophilic organism (Feller and Gerday, 2003).

True snow algae are regarded as psychrophilic organisms that thrive exclusively in snow (Komárek and Nedbalová, 2007; Leya, 2013). This classification is valid for some algae of the genus *Chloromonas* such as *Cr. pichinchae* (Hoham, 1975a), *Cr. tughillensis*, and *Cr. chenangoensis* (Hoham et al., 2008). However, other snow isolates such as *Cr. rosae* v. *psychrophile* (Hoham et al., 2008), *Cr. arctica* (Barcytė et al., 2018a) and *Cr. svalbardensis* (Barcytė et al., 2018b) can be regarded as psychrotolerant. These algae, in contrast to true snow algae, can inhabit other substrates (e.g., soil, freshwater) (Stibal and Elster, 2005; Komárek and Nedbalová, 2007), nevertheless, under persistent cold conditions they can acquire psychrophilic adaptions (Cvetkovska et al., 2017).

The psychrophilic nature of *Cm. collina* is also supported by the presence of plastoglobuli in the chloroplast, which contains specialized proteomes and metabolomes to respond to abiotic stress (van Wijk and Kessler, 2017). In addition, stressors such as high doses of UV-B radiation (Tian and Yu, 2009) or sulfur deficiency (Mizuno et al., 2013) have been reported to cause an increase in starch grains. The latter was observed in old cells of *Cm. collina* similar to *Cr. arctica* (Barcytė et al., 2018a), but unlike this alga, *Cm. collina* cells retain their flagella only at low temperature. When *Cm. collina* cells are exposed to elevated temperatures, they lose their flagella, similar to observed in the snow algae *Chlainomonas kolii* and *Chlainomonas rubra* (Hoham, 1975b).

### The Transcriptome of *Chlorominima collina* Reflects Adaptations to the Antarctic Snow Environment

**FIGURE 10:**
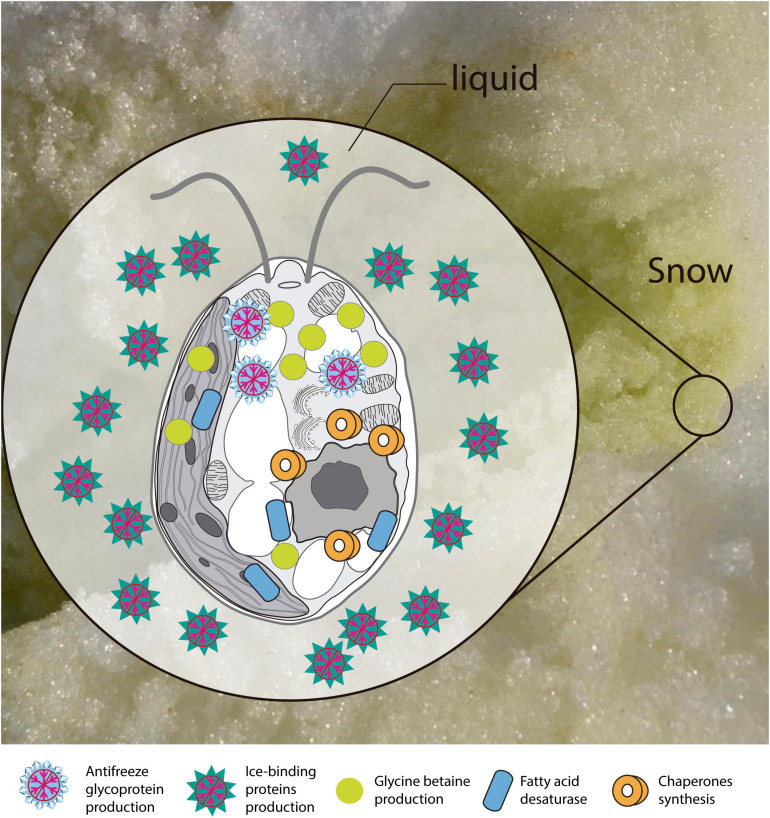
Scheme of the main transcriptional responses at low temperature. Putative proteins and enzymes present in the *Chlorominima collina* CCAP 6/1 transcriptome are represented.

Based on the functional annotation of the transcriptome, it is possible to identify the expression of the following groups of key genes: (i) stress-responsive genes, including genes that encode “heat shock proteins.” The expression of these genes is common in polar algae and cannot only be stimulated by heat, but also by cold (Liu et al., 2010, 2016; Kim et al., 2013). In addition, we identified CaM genes or “calmodulin,” whose expression is regulated by non-optimal temperature conditions, high UV-B radiation and salinity, conditions commonly found in Maritime Antarctica (He et al., 2017). Besides, the presence of genes of ABC transporters, linked to transport of metabolic intermediates and compounds for detoxification, as observed in other polar algae suggests a functional role in cold acclimatization (Mock et al., 2006; Liu et al., 2016; Poong et al., 2018). We also identified (ii) ice-active genes, such as genes encoding “antifreeze glycoprotein” and “ice-binding proteins” or IBPs, which have been found only in psychrophilic species (Oude Vrielink et al., 2016), particularly in snow species of the Chlamydomonadales (Leya, 2013). Therefore there are no homologs to IBPs in mesophilic species (Raymond et al., 2009; Dolhi et al., 2013). The novel IBPs discovered the Antarctic strain *Chlamydomonas* sp. CCMP681 showed effects on the inhibition of recrystallization and on the retention of brine in ice (Raymond et al., 2009), likewise the expression of IBP genes can also be regulated by heat (Jung et al., 2016a) and light stress (Gwak et al., 2014). The origin of these genes, whose resemblance to bacterial IBPs genes suggests the idea of a possible horizontal gene transfer (HGT), which can be facilitated by transposases (Raymond and Kim, 2012; Liu et al., 2016). Although some transposases were identified in the *Cm. collina* transcriptome, (TRINITY_DN1269; TRINITY_DN2803), evidence for HGT is still not conclusive. We also observe (iii) genes encoding cryoprotectants and fatty acids such as “Omega-3-fatty acid desaturase” involved in the biosynthesis of polyunsaturated fatty acids or PUFAs (Garba et al., 2017). Since PUFAs regulate membrane fluidity, their prevalence is regarded as one of the most common adaptations of psychrophilic organisms (De Maayer et al., 2014). Likewise, the presence of “Cytochrome P450, C-22 desaturase” genes can be indicative of sterol biosynthesis that also regulates the fluidity of the membrane (Brumfield et al., 2017). Furthermore, “Diacylglycerol acyltransferase” genes involved in the biosynthesis of triacylglycerol (TAG) were found, which is related to tolerance to freezing (Zienkiewicz et al., 2016; Tan et al., 2018). The presence of “Phytoene desaturase” genes may suggest an active synthesis of secondary carotenoids (Grünewald et al., 2000), whose antioxidant activity is also induced by cold (Gocheva et al., 2009; Lemoine and Schoefs, 2010). Other genes, such as those that encode “Betaine aldehyde dehydrogenase,” suggest the accumulation of betaine that reduce the freezing point in the cytoplasm (De Maayer et al., 2014). Thus, it is proposed that joint regulation of the above-mentioned genes allows *Cm. collina* (Figure 10) to thrive in Antarctic snow.

## Conclusion

Our study corroborates the suitability of using a polyphasic approach to identify and characterize snow alga *Cm. collina*. This new strain presents some typical morphological features of *Chloromonas*-like algae but differs from this genus by the unusual position of the nucleus, the shape of the chloroplast, the number of vacuoles and the small size of the cells. The phylogenies confirm that the new alga is not *Chloromonas* but forms an independent lineage, sister to other strains uncertainly assigned to *Chloromonas-Chlamydomonas*, forming a polar subclade within *Stephanosphaerinia*. Within the Chlamydomonadales, *Cm. collina* is proposed as a distinct taxon according to the comparisons with models of the secondary structures of the ITS2 rDNA. Based on these results, we propose a new Antarctic genus, *Chlorominima* with the species type *Cm. collina*. The integration of physiological and transcriptomic approaches revealed psychrophilic characteristics that reflect adaptations to the snow environment. Therefore, the description of *Cm. collina*, improves our knowledge on the diversity of snow algae within *Stephanosphaerinia.*

## Nomenclature

### Resource Identification Initiative

To take part in the Resource Identification Initiative, please use the corresponding catalog number and RRID in your current manuscript. For more information about the project and for steps on how to search for an RRID, please click here.

### Life Science Identifiers

Life Science Identifiers (LSIDs) for ZOOBANK registered names or nomenclatural acts should be listed in the manuscript before the keywords with the following format:

urn:lsid: < Authority > : < Namespace > : < ObjectID > [: < Version > ]

For more information on LSIDs please see Inclusion of Zoological Nomenclature section of the guidelines.

## Data Availability Statement

The datasets presented in this study can be found in online repositories. The names of the repository/repositories and accession number(s) can be found in the article/ Supplementary Material.

## Author Contributions

FEG sampled the algal material in the Antarctic, performed the isolation of the strain from cysts, and conducted the light microscopy and assisted in the electron microscopy studies. Also, carried out the physiological experiment and measurements for thermal threshold and transcriptomic analysis. PH supervised at the beginning the cultivation and maintenance procedures in the laboratory as well as the fluorescence techniques for growth rate measurement. AS and FEG with the help of the AUSTRAL-omics team and obtained the DNA sequences and performed the RNA extraction. MS-C performed the phylogenetic analyses and the sequence structure of the ITS2 rDNA. AS was in charge of the RNA library sequencing and transcriptional analysis. The first draft of manuscript was written by FEG and edited by IG. Finally, all authors contributed with inputs to the final stage of the manuscript.

## Conflict of Interest

The authors declare that the research was conducted in the absence of any commercial or financial relationships that could be construed as a potential conflict of interest.

## Footnotes

1 https://www.geneious.com

2 http://its2.bioapps.biozentrum.uni-wuerzburg.de

3 http://rna.tbi.univie.ac.at/cgi-bin/RNAWebSuite/RNAfold.cgi

4 http://www.ccap.ac.uk
