# Supplementary material for: Revealing the Characteristics of the Antarctic Snow Alga Chlorominima collina gen. et sp. nov. Through Taxonomy, Physiology, and Transcriptomics
Source: Front Plant Sci. 2021 Jun 7;12:662298. doi: 10.3389/fpls.2021.662298 (PMC8215615; doi:10.3389/fpls.2021.662298)
Supplement: Supplementary file 1 [file Data_Sheet_1.docx]

Supplementary Material

**
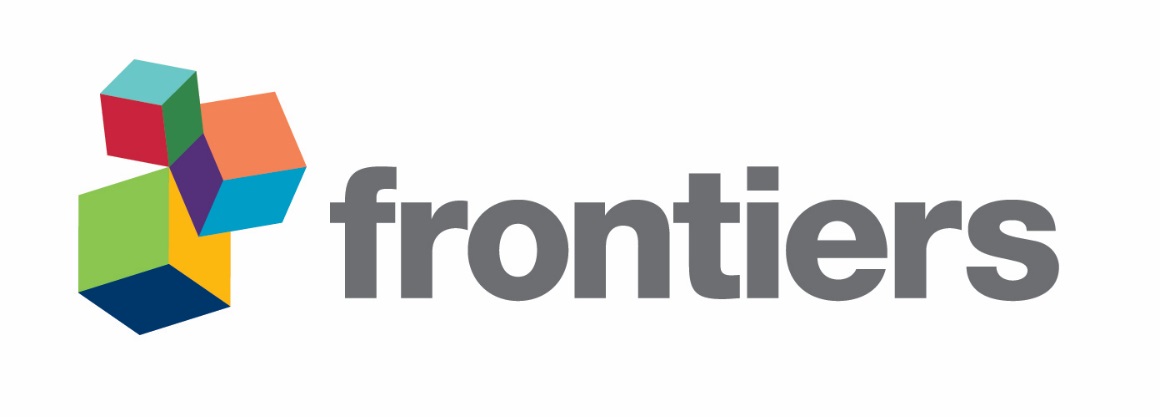
**

**
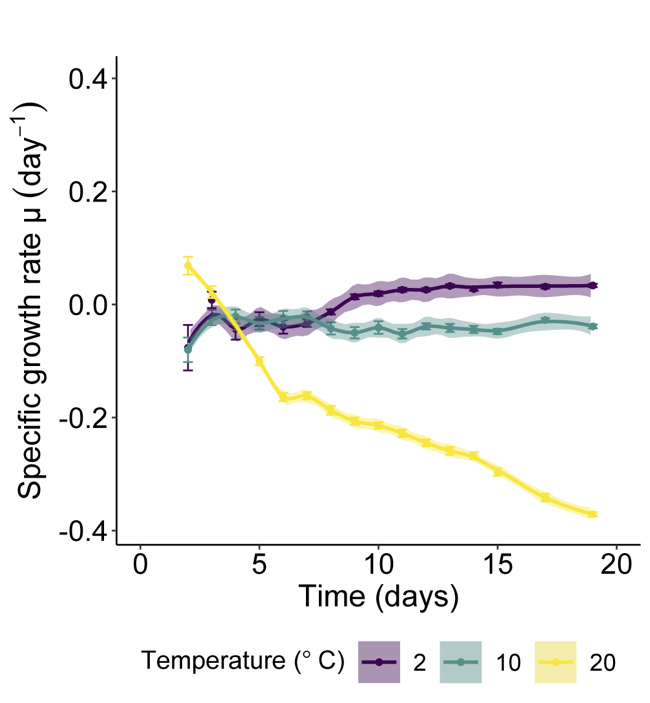
**

**Supplementary Figure 1.** Specific growth rate of *Chlorominima* *collina* CCAP 6/1 exposed to 2ºC, 10ºC and 20ºC. The lines show the trends, and the shading indicates the confidence intervals (CI). Each point is the mean of 24 replicates and the bars represent the standard error (SE).

**­Supplementary Table 1.** Summary of the effects of temperature and time on F_v_/F_m_ and RFU signals of *Chlorominima* *collina*.

| **Response variable** | **Model used** | **Predictor variable** | **Estimate** | **SE** | ***t/z* value** | ***p* value** |
| --- | --- | --- | --- | --- | --- | --- |
|  |  |  |  |  |  |  |
| **F_V_/F_M_** | Beta (GLMM) | Intercept  Temp.10  Temp.20  Time  Temp.10:Time | 0.3798  -0.1107  -0.1153  0.0086  -0.0325 | 0.0209  0.0221  0.0460  0.0016  0.0017 | 18.13  -5*.*00  - 2.51  5.21  -18.93 | < 0.0001 ***  < 0.0001 ***  0.0122 *  < 0.0001 ***  < 0.0001 *** |
|  |  | Temp.20:Time | -1.0246 | 0.0289 | -35.42 | < 0.0001 *** |
| **RFU** | Gamma  (GLMM) | Intercept  Temp.10  Temp.20  Time  Temp.10:Time  Temp.20:Time | 2.5082  0.2324  1.2128  0.0462.  -0.0818  -0.4023 | 0.0331  0.0553  0.0556  0.0048  0.0074  0.0076 | 75.747  4.203  21.810  9.558  -11.000  -52.936 | < 0.0001 ***  < 0.0001 ***  < 0.0001 ***  < 0.0001 ***  < 0.0001 ***  < 0.0001 *** |

**Signif. codes: 0 ‘***’ 0.001 ‘**’ 0.01 ‘*’ 0.05 ‘.’ 0.1 ‘ ’ 1**

**­Supplementary Table 2.** List of taxa used in this study, sisters to *Chlorominima collina* CCAP 6/1. Collection sites are specified.

|  | Genbank accession no. | |  |  |  |
| --- | --- | --- | --- | --- | --- |
| Taxon | 18S | rbcL | Collection site/Habitat | Reference |  |
| *Chloromonas* sp. KOPRI AnM0048 | JQ926737 |  | King Sejong Station, Antarctica/ freshwater | Choi,H.G. and Kang,S.H.; Unpublished |  |
|  |  |  |  |  |  |
| *Chloromonas* sp. KNF0032 | KU886306 |  | Dasan Station, Arctic/ freshwater | Jung et al. 2016a |  |
|  |  |  |  |  |  |
| *Chlamydomonas* sp. CCMP681 | EF106784 |  | Palmer Station, Antarctica/high intertidal | Raymond et al. 2009 |  |
|  |  |  |  |  |  |
| *Chloromonas* sp. CCCryo273-06 | HQ404890 |  | Ecology Glacier, Polish Arctowski Station, Antarctica/green snow | Leya,T. and Bey,T.D; Unpublished |  |
|  |  |  |  |  |  |
| Uncultured alga clone: Otu011 | LC371433 |  | Antarctica: Yukidori Valley/ red-snow samples | Segawa et al. 2018 |  |
| *Chlamydomonas* sp. CCMP681 |  | EU421061 & EU421062 | Palmer Station, Antarctica/High intertidal | Raymond et al. 2009 |  |
|  |  |  |  |  |  |
| *Chloromonas* sp. ANT1 |  | AF089834 | Petrel Island, Antarctica/ green snow | Devos et al. 1998; Raymond et al. 2009 |  |
|  |  |  |  |  |  |
